# Utility of Abdominal Cross-Sectional Imaging in Motor Vehicle Accidents in an Inner City Trauma Center: A Retrospective Cohort Study

**DOI:** 10.7759/cureus.73386

**Published:** 2024-11-10

**Authors:** Allen T Yu, Aliza S Gross, Alex L Huang, Jason Brody, Luis Suarez-Rodriguez, Susan Talbert, Raymond V Wedderburn, Kusuma Nio

**Affiliations:** 1 Trauma and Acute Care Surgery, Icahn School of Medicine at Mount Sinai, New York, USA; 2 Medicine, Icahn School of Medicine at Mount Sinai, New York, USA; 3 Trauma and Acute Care Surgery, Yale New Haven Hospital, New Haven, USA

**Keywords:** collision speed, ct of the abdomen and pelvis (ctap), intra-abdominal injury, motor vehicle collision, mvc

## Abstract

Background

The use of computed tomography (CT) of the abdomen and pelvis following motor vehicle collisions (MVCs) as standard diagnostic evaluation is widely accepted. However, the incidence of positive findings is low, and it is unknown which features increase the risk of having abdominal injuries.

Objectives

The aim of this study is to identify risk factors on presentation that are associated with positive CT findings.

Methods

A retrospective review of patients from January 2020 to January 2023 in a level II urban trauma center who were in MVCs was performed. Standard ACS TQIP metrics were recorded, as well as vehicle speed, CT findings, and presenting characteristics. Low-speed MVCs were considered to be ≤25 mph and high-speed MVCs were considered to be >25 mph.

Results

In 4,444 trauma activations, there were 738 (16.6%) MVCs: 310 (42.0%) were low-speed, 160 (21.7%) were high-speed, and 268 (36.3%) were unknown-speed. Twenty-nine patients had positive CT findings. There was a significant difference in positive CT findings in low-speed versus high-speed MVCs (1.9% vs 5.9%, p < 0.05). Multivariate analysis for positive CT findings revealed that high-speed and unknown MVCs (OR_adj_ 5.25 [95% CI 1.62-17.0] and OR_adj_ 3.84 [1.34-11.0], respectively) were significant risk factors for positive CT findings. The number needed to scan for a positive CT finding was 53 patients for low speed, 17 for high speed, and 19 for unknown speed.

Conclusion

Our data indicate that a high-speed MVC is a discrete risk factor for positive CT findings. More research is needed to determine if there are other clinical factors to ultimately create a set of criteria for abdominal imaging in trauma.

## Introduction

Motor vehicle collisions (MVCs) are a major cause of morbidity and mortality in the United States, accounting for approximately 40,000 deaths annually [1]. CT scanning is often used in the emergency room setting to rapidly assess for internal injuries in blunt trauma patients, including MVC patients, with high sensitivity [2]. This is especially important for intra-abdominal injuries, as physical examinations have been shown to be an unreliable indicator of abdominal injury in blunt trauma patients [2-4]. Because of this, current American College of Radiology Appropriateness Guidelines state whole-body CT (WBCT), including CT of the abdomen and pelvis (CTAP), may be warranted in hemodynamically stable patients with blunt trauma including MVC over 35 mph, MVC with rollover or passenger ejection, motorcycle accidents, bicycle accidents, MVC-pedestrian collision, or a fall from a height of >15 feet [5]. However, even blunt trauma patients who do not meet these criteria may reflexively undergo a CTAP, as there is no clinically validated decision-making pathway for abdominal CT, unlike cervical spine or head imaging [6,7]. The liberal use of CT scanning is associated with increased cost and radiation exposure for patients, and, thus, determining which patients truly require CT and applying appropriate criteria in imaging is of great importance [8,9].

The literature is unclear as to what clinical factors put patients at high versus low risk for intra-abdominal injury. For example, several studies have found that hemodynamically stable blunt trauma patients with a negative abdominal examination and normal mental status had very low rates of positive CT findings, with no increase in mortality or change in clinical management as a result of injuries [10-12]. However, other studies have found that MVC patients had significant rates of intra-abdominal injury even in the absence of a positive physical examination, although these studies varied in the severity of the types and speeds of the crashes included [2-4]. Additionally, the impact that vehicle speed has on the risk of intra-abdominal injury remains understudied, and there is no official guidance as to whether victims of low-speed MVCs truly require CTAP [13-15].

To address this gap, we performed a study at a trauma center based in a dense urban inner city where vehicle speeds are restrained by a 25-mph speed limit, and the number of pedestrian and bicycle accidents is greater [16]. The aim of this study was to determine if there is an MVC speed at which patients are at higher risk for intra-abdominal injuries, as well as to identify other risk factors associated with positive CT findings, to distinguish a population of MVC patients in whom the utility of a CTAP may be limited.

This work was presented at the Academic Surgical Congress on February 8, 2024, in Washington, DC.

## Materials and methods

We conducted a retrospective review of patients from January 2020 to January 2023 in a level II urban trauma center who were in MVCs. Data were extracted from the National Trauma Registry of the American College of Surgeons (NTRACS) trauma database maintained by the database’s manager in the institute, which has 100% capture of all trauma activations, which were all included as the patient base in this study. Exclusion criteria included patients who were hemodynamically unstable, had a Glasgow Coma Scale (GCS) score of <12, or were <18 years of age. The standard American College of Surgeons Trauma Quality Improvement Program (ACS TQIP) registry metrics were used, which included presenting vital signs, GCS score, injury severity scale (ISS), and mechanism of trauma, and additional variables were abstracted from the medical record outside of the program, including vehicle speed given by the patient or bystanders, physical examination findings, and CT findings. Low-speed MVCs were considered to be ≤25 mph and high-speed MVCs were >25 mph. Missing speed data were categorized as unknown.

Patient demographics and unadjusted outcomes were calculated using the chi-square test, with Yate’s continuity correction for categorical variables and Student’s t-test or Wilcoxon test where appropriate for continuous variables. A multivariable generalized logistic regression model was used to evaluate the risk of having a CT scan with positive findings and a positive abdominal examination, adjusting for clinically relevant covariates, as well as those found to be significant on univariate analysis. These covariates included age, sex, BMI, ISS, abdominal examination, and MVC category. Statistical analysis was performed using R version 4.3.2 (R Foundation for Statistical Computing, Vienna, Austria). This retrospective study was approved by the Institutional Review Board (IRB# 23-00580). STROBE (Strengthening the Reporting of Observational Studies in Epidemiology) guidelines were used when preparing the manuscript.

## Results

Patient population

**Figure 1:**
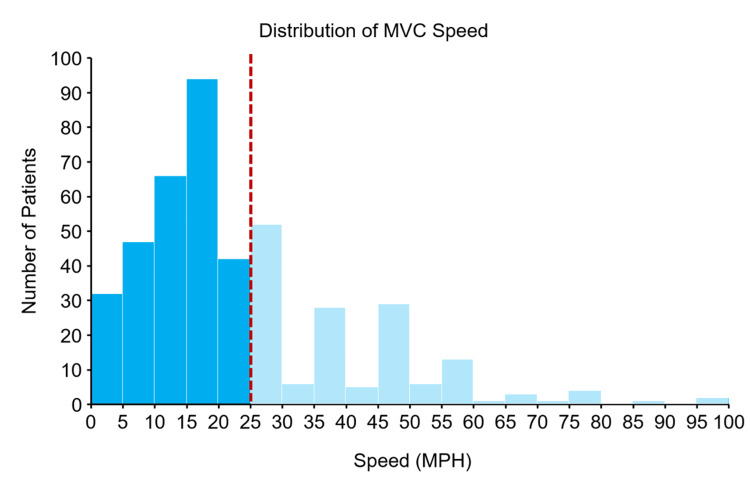
Distribution of MVC speed MPH, miles per hour; MVC, motor vehicle collision

A total of 4,444 trauma activations occurred between January 2020 and January 2023, 738 of which involved MVCs. The patient population was predominantly male, 567 (76.8%), with an average body mass index (BMI) of 26.8 ± 6.0 kg/m2 and an ISS of 4.5 ± 4.8. Initial vitals on presentation included an average heart rate (HR) of 86.2 ± 17.5, systolic blood pressure of 139.9 ± 21.7, and diastolic blood pressure of 86.6 ± 16.6. MVC categories were largely evenly distributed, with 193 (26.2%) car versus car, 218 (29.5%) bicycle versus car, 170 (23.0%) pedestrian versus car, and 157 (21.3%) motorcycle or scooter versus car. A total of 29 (4.0%) patients had positive CTAP findings, and 123 (16.7%) had positive abdominal examinations (Table 1). The average speed the car involved in the collision was traveling was 25.9 ± 16.6 mph. There was an appropriately right-skewed distribution in MVC speed, as shown in Figure 1.

**Table 1:** Patient demographics CTAP, CT of the abdomen and pelvis

Characteristic	n = 738	% or SD
Age (years)	39.9	17.7
Sex		
Male	567	76.8
Female	171	23.2
Body mass index (kg/m^2^)	26.8	6
Injury severity scale	4.5	4.8
Heart rate	86.2	17.5
Systolic blood pressure	139.9	21.7
Diastolic blood pressure	86.6	16.6
Collision speed (mph)	25.9	16.6
Motor vehicle collision category		
Car	193	26.2
Bicycle	218	29.5
Pedestrian	170	23
Motorcycle or scooter	157	21.3
Positive CTAP finding	29	4
Positive abdominal examination	123	16.7

Of the 29 positive CTAP findings, 8 (27.6%) involved the kidney, 7 (24.1%) were hematomas, 5 (17.2%) involved bowel, 5 (17.2%) were liver injuries, 3 (10.3%) were spleen injuries, and 1 (3.4%) was a bladder injury (Table 2). A total of five (17.2%) patients required intervention, of whom two required exploratory laparotomies, one for free air and one for intraperitoneal bladder injury. The other three patients underwent angioembolization.

**Table 2:** List of positive imaging findings

Type of injury	n = 29
Kidney	8 (27.6%)
Hematoma	7 (24.1%)
Bowel	5 (17.2%)
Liver	5 (17.2%)
Spleen	3 (10.3%)
Bladder	1 (3.4%)

Stratification of patients by collision speed

We found that when comparing the two-speed groups, a positive CTAP finding was seen at a significantly higher rate in the high-speed group than in the low-speed group (6, 1.9% vs 9, 5.8%, p = 0.028). In addition, other clinical attributes such as ISS and HR were also statistically significant. Naturally, the MVC category had differences as well, with car versus car accidents predominantly being high-speed and bicycle or pedestrian versus car predominantly being low-speed (Table 3).

**Table 3:** Stratification of patients by 25-mph collision speed *p < 0.05, **p < 0.01, ***p < 0.001 CTAP, CT of the abdomen and pelvis

Total population, n = 738	≤ 25 mph, n = 310	> 25 mph, n = 160	Unknown mph, n = 268	p-Value
Age (years)	38.8 (17.2)	38.2 (16.7)	42.2 (18.8)	0.027*
Male	249 (79.6)	124 (77.5)	198 (73.1)	0.605
Body mass index (kg/m^2^)	26.9 (5.8)	27.2 (6.4)	26.3 (6.0)	0.338
Injury severity scale	3.8 (4.4)	4.4 (4.1)	5.4 (5.6)	0.001**
Heart rate	83.5 (14.8)	87.1 (16.9)	88.8 (20.1)	0.001**
Systolic blood pressure	139.7 (21.7)	139.6 (20.7)	140.2 (22.4)	0.956
Diastolic blood pressure	86.1 (16.8)	85.6 (18.0)	87.9 (15.4)	0.278
Positive CTAP finding	6 (1.9)	9 (5.8)	14 (5.2)	0.028*
Positive abdominal examination	54 (17.4)	30 (18.8)	42 (15.7)	0.721
Motor vehicle collision category				
Car	35 (11.3)	91 (56.9)	67 (25.0)	<0.001***
Bicycle	108 (34.8)	24 (15.0)	86 (32.1)	<0.001***
Pedestrian	78 (25.2)	17 (10.6)	28 (10.4)	<0.001***
Motorcycle or scooter	89 (28.7)	28 (17.5)	40 (14.9)	0.011*

As not all patients or bystanders could recall the speed of the vehicle, we made a separate category for unknown speed. The predominant MVC category in this category was bicycle versus car (86 patients, 32.1%), and 14 patients (5.2%) had positive CTAP findings in this group. Interestingly, this group also had the highest ISS and initial HR compared to the low-speed and high-speed groups and had the lowest proportion of patients with a positive abdominal examination (42 patients, 15.7%) (Table 3).

Univariate and multivariate analysis of positive CT findings

When we assessed if the patients who had positive CTAP findings had differences in their baseline attributes, we observed that on univariate analysis, only high-speed collisions and unknown-speed collisions were risk factors for having positive CTAP findings (OR 3.02 [1.06-8.64], p = 0.039 and OR 2.80 [1.06-7.37], p = 0.038, respectively). While the category of MVC was not a significant risk factor for positive CTAP findings, pedestrian versus car was approaching significance (OR 2.35 [0.79-7.02], p = 0.126) (Table 4).

**Table 4:** Univariate and multivariate analysis of positive imaging findings *p < 0.05, **p < 0.01, - no value CTAP, CT of the abdomen and pelvis

CTAP findings, n = 29	Univariate, OR (95% CI)	p-value	Multivariate, OR (95% CI)	p-Value
Age (years)	1.01 (0.99-1.03)	0.288	1.01 (0.98-1.03)	0.42
Sex (male vs female)	1.06 (0.44-2.52)	0.9	0.99 (0.38-2.62)	0.992
Body mass index (kg/m^2^)	1.00 (0.94-1.07)	0.934	1.00 (0.94-1.06)	0.911
Positive abdominal examination	1.91 (0.83-4.41)	0.131	2.36 (0.98-5.65)	0.054
Speed				
≤25 mph	1.00 (ref)	-	1.00 (ref)	-
>25 mph	3.02 (1.06-8.64)	0.039*	5.25 (1.62-17.0)	0.006**
Unknown speed	2.80 (1.06-7.37)	0.038*	3.84 (1.34-11.0)	0.012*
Motor vehicle collision category				
Car	1.00 (ref)	-	1.00 (ref)	-
Bicycle	1.06 (0.32-3.54)	0.919	1.82 (0.49-6.81)	0.371
Pedestrian	2.35 (0.79-7.02)	0.126	3.08 (0.91-10.4)	0.072
Motorcycle or scooter	2.02 (0.65-6.23)	0.226	3.84 (1.14-12.9)	0.030*

To determine if high-speed or unknown-speed collisions were independently associated with positive CTAP findings, we performed a multivariable analysis with the outcome variable set as positive CTAP findings, adjusting for age, sex, BMI, positive abdominal examination, speed, and MVC category. We found that high-speed collisions and unknown-speed collisions were independent risk factors for having positive CTAP findings (OR_adj_ 5.25 [1.62-17.0], p = 0.006 and OR_adj_ 3.84 [1.34-11.0], p = 0.012, respectively). Interestingly, the only category of MVC that was significant was motorcycle or scooter versus car (OR_adj_ 3.84 [1.14-12.9], p = 0.030), with pedestrian versus car approaching significance (OR_adj _3.08 (0.91-10.4), p = 0.072). A positive abdominal examination was also approaching statistical significance (OR_adj _2.36 [0.98-5.65], p = 0.054).

Univariate and multivariate analysis of positive abdominal findings

We also performed additional analysis to determine if positive abdominal examination findings were a reliable indicator of positive CTAP findings. On univariate analysis, there were no significant associations with positive abdominal examinations. On multivariate analysis, no covariates were significantly associated with a positive abdominal examination, but interestingly a collision on a motorcycle or scooter was approaching significance when compared to a car versus car collision (OR_adj_ 0.89 [0.29-1.03], p = 0.062) (Table 5).

**Table 5:** Univariate and multivariate analysis of positive abdominal examination *p < 0.05, **p < 0.01, - no value

Positive abdominal examination, n = 126	Univariate, OR (95% CI)	p-Value	Multivariate, OR (95% CI)	p-Value
Age (years)	1.00 (0.99-1.01)	0.665	1.00 (0.99-1.01)	0.637
Sex (male vs female)	1.22 (0.79-1.89)	0.378	0.96 (0.59-1.57)	0.87
Body mass index (kg/m^2^)	1.21 (1.00-1.06)	0.097	1.03 (0.99-1.06)	0.103
Speed				
≤25 mph	1.00 (ref)	-	1.00 (ref)	-
>25 mph	1.09 (0.67-1.79)	0.721	0.93 (0.53-1.63)	0.788
Unknown speed	0.88 (0.57-1.37)	0.574	0.87 (0.54-1.38)	0.549
Motor vehicle collision category				
Car	1.00 (ref)	-	1.00 (ref)	-
Bicycle	0.71 (0.43-1.17)	0.178	0.72 (0.40-1.29)	0.267
Pedestrian	0.82 (0.48-1.39)	0.459	0.54 (0.49-1.60)	0.693
Motorcycle or scooter	0.62 (0.35-1.10)	0.104	0.89 (0.29-1.03)	0.062

## Discussion

This study sought to determine if MVC speed was associated with positive CTAP findings, as well as if there were other risk factors for intra-abdominal injury in MVCs. We determined that patients in high-speed accidents greater than 25 mph were significantly more likely to have positive CTAP findings than patients in low-speed accidents. After adjusting for potential confounding variables, these associations remained significant. Patients with accidents of unknown speed were trending towards significance. We found that in our cohort, the number needed to scan for a positive CT finding were 53 patients for low-speed, 17 for high-speed, and 19 for unknown-speed accidents.

Positive abdominal examinations as a predictor of positive CTAP findings also approached significance on multivariate analysis, with a p-value of 0.054. The literature surrounding abdominal examinations as a predictor of intra-abdominal injury is heterogeneous. Studies have found that 7.1-16% of patients with negative abdominal examinations end up having a positive CTAP [2-4,15,17]. However, other studies suggest that CT does not provide information that changes management in stable patients with negative abdominal examinations and that observation and clinical judgment may be sufficient to screen for significant intra-abdominal injury [11,18,19]. Some studies have shown other clinical factors that may be used in conjunction with abdominal examinations to predict the likelihood of positive CTAP findings, including GCS, urinalysis, hematocrit, and the presence of other severe injuries [10,20]. Mechanism of injury was notably not included in these clinical models; therefore, it is unknown if speed or type of MVC has any bearing on the utility of these clinical prediction tools.

We hypothesized that crash type would make a difference, as bicycle versus car accidents are associated with more severe injuries than car versus car accidents [21]. Additionally, higher-speed pedestrian versus car accidents are associated with more severe injuries and mortality [22,23]. However, the impact of crash mechanism on intra-abdominal injuries specifically is understudied. We found on multivariate analysis that only motorcycle or scooter versus car was an independent risk factor for a positive CTAP finding. This is likely because 75% (six out of eight) of patients with positive CTAP findings in this category had MVCs that were of high or unknown speed.

A limitation of this study was that accident speeds were self-reported by patients or witnesses at triage, and thus the speeds recorded may not all be the actual speeds of the accidents. However, clinical decision-making must be based on the information we receive from the patient in the emergency room. Thus, studying self-reported crash speed may be more relevant and applicable to real clinical contexts where the patient may not know the exact speed. In addition, our results are largely specific to urban areas, given the high concentration of vehicles and traffic lights. Thus, a larger scale study to confirm the generalizability of the 25-mph cutoff is needed and to resolve the incidence of CTAP findings by binned speed.

Additionally, other crash characteristics that we did not study may be important in determining the risk of intra-abdominal injury. Studies have found that prolonged extrication, seatbelt use, placement within the car, and rollover may increase the odds of positive whole-body trauma CT and CTAP specifically [13,14]. In pedestrian and cyclist accidents, the type of vehicle striking the patients may impact the risk of severe injury and mortality [21-23]. This study did not stratify MVCs by these additional variables, but future studies may benefit from examining how these variables influence the relationship between MVC speed and intra-abdominal injuries. Clinical factors such as co-morbidities and anticoagulation use could possibly be relevant as well and are important areas of future investigation. While patients on anticoagulation have been shown in some studies to be associated with worse outcomes in brain injury, the relationship between anticoagulation and intra-abdominal injuries is not well characterized [24].

Based on these findings, the routine use of CTAP in select low-speed MVC patients could be reconsidered. While the number needed to scan is 53 patients for a positive CTAP finding, only one out of 310 low-speed MVC patients required intervention. Thus, 333 patients need to be scanned to find an intervenable injury. However, the cost of missing this injury could be devastating, and clinical judgment must be exercised when choosing to forgo abdominal imaging. Additional observation or strict return precautions should be given to those patients.

## Conclusions

Overall, our retrospective study of an inner city trauma center showed that there is an increased risk of positive CTAP findings in patients who were in MVCs reported as greater than 25 mph and of unknown speed, which remained significant after multivariate analysis. The only other factor we found to be associated with positive CTAP findings was motorcycle or scooter versus car, while age, gender, and BMI were not significantly associated with increased odds of intra-abdominal injury. A positive abdominal examination trended towards increased odds of a positive CTAP but was not statistically significant. More research is needed to determine if there are other clinical factors that may put low-speed MVC patients at increased risk of intra-abdominal injury to ultimately create a set criterion for abdominal imaging in trauma.
